# Risk of ciguatoxins is shaped by *Gambierdiscus* community structure

**DOI:** 10.1371/journal.pone.0341899

**Published:** 2026-01-29

**Authors:** Kirsty F. Smith, Lesley L. Rhodes, Belinda Curley, Arjun Verma, Gurjeet Kohli, David Tim Harwood, James Sam Murray, Jérôme Viallon, Hélène Taiana Darius, Mireille Chinain, Teina Rongo, June Hosking, Phoebe A. Argyle, Jacqui Stuart, Shauna A. Murray

**Affiliations:** 1 Cawthron Institute, Nelson, New Zealand; 2 Laboratory of Marine Biotoxins, Institut Louis Malardé, UMR-241 EIO (Ifremer, ILM, IRD, UPF), Tahiti, French Polynesia; 3 NSW Department of Primary Industries, Port Stephens, New South Wales, Australia; 4 Kōrero O Te `Ōrau, Rarotonga, Cook Islands,; 5 Mauke, Cook Islands; 6 University of Technology Sydney, School of Life Sciences, Sydney, New South Wales, Australia; Stockholm University, SWEDEN

## Abstract

Ciguatoxins (CTXs) are produced by marine microbial eukaryotes (*Gambierdiscus/Fukuyoa*, Dinophyta: Alveolata) that live epiphytically on macroalgae and other substrates. When CTXs accumulate in seafood they cause Ciguatera Poisoning (CP), which affects ca. 20–50,000 people p.a. and is likely worsened by climate change. CTXs in fish are highly variable with diet, ecology, size, age and phylogeny. Chemical identification of CTXs is difficult, and while detection of *Gambierdiscus* is simple, often no clearly CTX-producing *Gambierdiscus* are known from CP sites. Here, we coupled custom deep metabarcoding with quantitative PCR, CTXs in both *Gambierdiscus* and a sentinel fish (*Ctenochaetus striatus*) with limited home range, in the Cook Islands, an endemic CP area. Using neuroblastoma cell assays and liquid chromatography-tandem mass spectrometry, CTXs were present in fish species from six families. Dinoflagellate α-diversity was dominated by nine *Gambierdiscus/Fukuyoa* spp., the highest diversity yet reported from a single location. Sites where the rare species, *G. polynesiensis,* were detected on artificial substrates and macroalgae were more closely aligned with concentrations of P-CTX3B in *C. striatus*, unrelated to fish age or size, than with overall *Gambierdiscus* abundance. Of newly isolated *Gambierdiscus* strains, only the three *G. polynesiensis* produced P-CTXs. We show rare *Gambierdiscus* spp. and sentinel fish can map site-specific CP risk to address a growing climate change-related public health threat.

## Introduction

Ciguatera Poisoning (CP) is the most common, non-bacterial seafood-associated illness globally, with 20,000–50,000 cases reported annually, mainly from tropical and subtropical regions [1]. It is caused by ciguatoxins (CTX), produced by microbial eukaryotes (*Gambierdiscus* and *Fukuyoa* species; class Dinophyceae), which live epiphytically on macroalgae and dead coral. Consumption of these microorganisms by marine animals, in particular herbivorous fish, results in toxin accumulation. When CTXs accumulate in seafood species and are consumed by humans they can cause serious illness. CTXs can be highly toxic, for example, Pacific CTX1B has an extremely low LD_50_ of 0.25 µg/kg in mice by intraperitoneal injection [2] and can result in death or disablement in humans [3].

The South Pacific region is a CP hotspot, with cases increasing by 60% during a 20-year period [4,5]. Multiple factors can increase the abundance of *Gambierdiscus* and CTXs, and therefore the risk of CP, including seawater temperature, increased nutrient loading in lagoons, coral bleaching and reef disturbance by cyclones and predators (e.g., crown of thorns starfish) which leads to increased availability of substrates for *Gambierdiscus* spp. [6,7]. Climate impacts such as increasing temperatures (i.e., some *Gambierdiscus* species grow optimally at higher temperatures than normally recorded) [8] and alterations to oceanic current circulation (i.e., the transport of *Gambierdiscus* and contaminated fish to new areas); [9,10] can also affect CP incidence. CP is therefore a human disease likely worsened by climate change, requiring a ‘One Health’ approach to its management and mitigation [11].

Understanding the localised risk of CP is crucial to prevent illness but to date this has been extremely challenging. While the detection of *Gambierdiscus/Fukuyoa* spp. is potentially simple and inexpensive via microscopy or molecular methods [12], species differ markedly from one another in CTX-like activity in bioassays but the confirmation of chemically characterised CTXs using liquid chromatography-mass spectrometry (LC-MS) has not been established for most species [13–17]. Contradictory reports of the toxin production and toxicity of *Gambierdiscus/Fukuyoa* spp., have also been found using LC-MS/MS and cell-based toxicity assays [18–21] and in many regions no confirmed CTX-producing *Gambierdiscus* spp. have been found from environmental surveys, despite CP being endemic to the area [9]. Additionally, high trophic level fish, which are known to contain the highest levels of CTXs, often have large home ranges, meaning that detection of CTXs in one fish may not be indicative of CP risk from other fish at the same location [22].

The Cook Islands is a Pacific small island developing state (SIDS) that has amongst the world’s highest incidences of CP, with an estimated rate of 1058 reports per 10,000 population p.a. (1989–2006) [6,23]. CP is not preventable by seafood storage or cooking methods, and CTXs are odorless, tasteless and heat stable [3]. Symptoms of CP are neurological, gastro-intestinal and cardiovascular [3] and this has led to a reduction in reef fishing and a dietary shift to meat protein, causing population health declines and associated negative impacts [6,23]. The detection of CTXs in fish and invertebrates also relies on costly technology and expertise, which are inaccessible to SIDS that have the greatest incidence rates of CP. Given increasing risk factors, it is of increasing importance that causative factors of CP be understood at a local scale in SIDS so that CP detection and prevention can be put in place.

## Materials and methods

### Ethics statement

Samples were collected under the Ministry of Marine Resources, Cook Islands, research permit 26−14.

### Cook islands field sampling

**Fig 1:**
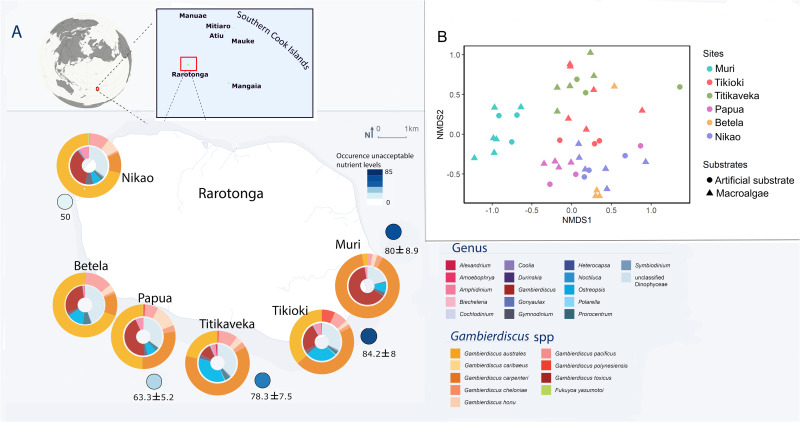
Sampling sites at Rarotonga (Cook Islands) and water quality data. A. Percentage of occasions with unacceptable nutrient concentrations, as dissolved inorganic nitrogen and dissolved reactive phosphorus) from monthly monitoring from 2007 to 2011, based on international standards for human and coral reef health (MMR, 2012) to provide a baseline indication of the water quality at each site. ND = no data. Relative abundance of Dinophyceae reads at the genus and species level using high-throughput sequencing metabarcoding. Outer circles show reads for species of *Gambierdiscus* and inner circles show reads for all Dinophyceae. Classification and read numbers are available in S3 Table. B. Dinoflagellate species (α) diversity calculated using non-metric multidimensional scaling (nMDS) showing similarities in Dinophyceae species presence/absence using high-throughput sequencing metabarcoding between sampling locations and methodologies. Maps were created using QGIS (v3.42.2), with design refinement in Adobe Illustrator.

Macroalgal, artificial substrate and fish samples were collected at six sites in the lagoon surrounding Rarotonga, Cook Islands, between the 2^nd^-14^th^ November 2014 (Fig 1A). At each site, six macroalgal samples were collected (S1 Table) and preserved for metabarcoding analyses as previously described [12]. Additionally, from each sample, a 50 mL subsample was taken for live cell isolations. Samples were also collected using an artificial substrate method (100 mm × 150 mm) as described in Smith et al. [24]. At each site, six artificial substrates were deployed for 48 hours. Three artificial substrates were preserved in ambient seawater with Lugol’s solution for microscopic analyses and three artificial substrates were preserved in ca. 40 mL of laboratory-made nucleic acid preservation solution as described in [25]. Additional artificial substrate samples were also collected during a follow-up field survey in Rarotonga in December 2022 from sites Muri, Titikaveka, and Nikao. Water quality data (shown as percentage of occasions of unacceptable nutrient concentrations) was sourced from Ministry of Marine Resources (MMR; Government of the Cook Islands) monthly monitoring data from 2007 to 2011. Acceptable nutrient levels (dissolved inorganic nitrogen and dissolved reactive phosphorous) are based upon international standards for human and coral reef health [26].

### Fish sample collection and otolith analyses

Two fish species, *Ctenochaetus striatus* (Maito/striated surgeonfish) and *Chrysiptera glauca* (Grey demoiselle/damselfish), were used as potential sentinel species to examine the spatial relationship between the occurrence and abundance of *Gambierdiscus* spp. and levels of CTXs in localised fish populations. These species have a wide distribution in the Pacific, a relatively small home range and feed directly off the substrate. *Ctenochaetus striatus* has a feeding home range <0.30 km^2^, is a detrital aggregate brusher removing microorganisms, organic detritus and inorganic sediment, and may also act as a secondary herbivore [27]. This species is a well-known vector of CTXs [28] and has been linked to cases of CP in Rarotonga [6,29]. The damselfish *C. glauca* is not targeted by fishers and has not, to our knowledge, been tested for CTXs previously. However, if CTXs were detected in this species it would expect to exhibit a strong relationship with *Gambierdiscus* distribution and therefore CP risk within a region as *C. glauca* has a very small home range (m^2^) and is algivorous with a grazer sucker feeding mode [30]. Several other species, with a range of diets, were also sampled to gain insight into the prevalence of CTX in fishes in Rarotonga (S2 Table). *Ctenochaetus striatus* and *C. glauca* specimens were collected using an anaesthetic (5% clove oil in ethanol) and a hand-spear. All other species were collected via spear fishing by Cook Islands MMR fisheries staff. For each fish sample a small subsample of flesh tissue (0.5 g) was collected and immediately frozen until DNA extraction and identification using phylogenetic analyses as described below. The standard and total length of each fish was measured to the nearest mm. Sagittal otoliths were extracted on the day of capture or fish were frozen and otoliths extracted later in the laboratory. Otoliths were washed, air dried, and stored in Eppendorf tubes. All otoliths were weighed, sectioned and read by Fish Ageing Services Pty Ltd Victoria, Australia (FAS) to obtain annual age estimates. Additional *C. striatus* individuals were collected during a follow-up field survey in Rarotonga in December 2022 from sites Muri, Titikaveka, and Nikao.

### *Gambierdiscus* isolation, culture maintenance and microscope counts

Single cells of *Gambierdiscus* were isolated from live macroalgal samples using capillary tubing attached to a modified Pasteur pipette as previously described in Rhodes et al. [31]. Cultures were maintained in f/10 medium, 25°C, ∼60 µmol photons m^-2^s^-1^, 12:12 light:dark cycle at the University of Technology Sydney or the Cawthron Institute Culture Collection of Microalgae.

Fixed artificial substrate samples for enumeration were gently homogenised to dislodge cells from the substrates. Subsamples (10 mL) were taken and examined using inverted light microscope (LM) and Utermöhl chambers. The total number of *Gambierdiscus* cells, identified to the genus level, were enumerated to estimate the total number of cells per artificial substrate.

### DNA extractions for environmental samples, *Gambierdiscus* cultures and fish tissue samples

DNA from preserved environmental samples and cultures established from live cell isolations were extracted as described in Smith et al. [24]. DNA was extracted using Power Soil® DNA isolation kits (Qiagen, Valencia, CA, United States) following the manufacturer’s protocol. Tissue samples from fish were extracted using G-spin Total DNA extraction kits (using the animal tissue protocol; Intron, Gyeonggi-do, South Korea).

### *Gambierdiscus* isolate and fish phylogenetic analyses

The D8-D10 fragment of the large subunit ribosomal DNA (LSU rDNA) was amplified from *Gambierdiscus* cultures using primers D8F (5'-CGGGAAAAGGATTGGCTCT-3') and D10R (5'-ATAGGAAGAGCCGACATCG-3') [32]. A 600 bp section of the mitochondrial cytochrome oxidase I (COI) gene was amplified from fish samples using the primers FishF2 (5'-TCGACTAATCATAAAGATATCGGCAC-3') and FishR1 (5'-TAGACTTCTGGGTGGCCAAAGAATCA-3') [33]. The PCR amplifications were carried out in 50 μL reaction volumes containing MyTaq™ 2x PCR master mix (Bioline, MA, USA), both forward and reverse primers (0.4 μM final concentration) and template DNA (ca. 50–150 ng). Thermocycling conditions for LSU rDNA region were an initial denaturing step of 95°C, 2 min, 35 cycles of 95°C, 30 s, then 54°C, 30 s and 72°C, 60 s with a final extension step of 72°C, 5 min. Thermocycling conditions for the COI gene were an initial step of 2 min at 95°C followed by 35 cycles of 0.5 min at 94°C, 0.5 min at 54°C, and 1 min at 72°C, followed in turn by 10 min at 72°C. Sanger sequencing was carried out by an external contractor (Genetic Analysis Services, University of Otago, Dunedin). Bayesian analyses were carried out in Geneious using MrBayes 3.1.2 [34] as described previously [31].

### Metabarcoding PCR conditions, Illumina™ MiSeq analysis and bioinformatics

DNA extractions from environmental samples were amplified using the primers D1R (5'-ACCCGCTGAATTTAAGCATA-3') and 305R (5'-TTTAAYTCTCTTTYCAAAGTCC-3') targeting the D1-D2 region of the LSU rDNA region as described in Smith et al. [24]. PCR was carried out in 50 μL reaction volumes containing 25 μL of MyFi™ Mix (Bioline, MA, USA), 0.4 μM of both forward and reverse primers, and ca. 50–150 ng of template DNA. Thermocycling conditions were 94°C for 2 min, followed by 35 cycles of 94°C for 20 s, 55°C for 10 s, 72°C for 45 s, and a final extension of 72°C for 5 min. The samples were sent to Auckland Genomics (University of Auckland, New Zealand) for further processing. The samples were normalised to 5 ng μL^−1^. Libraries were prepared using the Illumina™ two-step PCR amplicon library preparation method and sequenced using an Illumina™ MiSeq sequencer with 2 × 250 base paired-end reads. All data generated was quality checked by Auckland Genomics using the following processes: FastQC, FastQscreen, and SolexaQA [35]. The raw sequences were deposited in the NCBI sequence read archive under the accession number: PRJNA1315151. Further bioinformatic analyses were carried out in Mothur v1.37.6 [36] as described previously [24]. To align and classify the reads, we created a custom dinoflagellate reference sequence database for each gene region as previously described [24].

Alpha diversity of each site was calculated using the Shannon diversity index using the *diversity* function in the R package *vegan* v.2.5–7 (http://www.r-project.org) [37]. Differences in diversity were assessed by a one-way analysis of variance (ANOVA) and differences between each site were identified by a Tukey Honest Significant Difference test. Non-metric multidimensional scaling (nMDS) was used to visualize the community structure. Statistical differences in community structure been sites and sampling methods were tested using Analysis of Similarity (ANOSIM) in the *vegan* v. 2.5–6 R package [37]. Figures were constructed in R using the package ggplot2 [38].

### Quantitative polymerase chain reaction (qPCR) analyses

The qPCR assay targeting *G. polynesiensis* was run in triplicate, using 5 µL reaction volumes containing 2.5 µL iTaq UniverSYBR Green SMX 2500®, 1.1 µL nuclease free water, 0.4 µM of each primer (final concentration) [39], and 1 µL of DNA template. Samples were run both undiluted and diluted 1:10 to detect evidence of PCR inhibition in the environmental samples. All reagents were aliquoted and plate preparation was conducted using an epMotion®5075l Automated Liquid Handling System. Negative control samples were run for detection of contamination. The qPCR was performed using the BIORAD CFX384 Touch™ Real-Time PCR Detection System™ using the thermal cycling conditions of 95°C for 2 min followed by 40 cycles at 95°C for 10 s, annealing for 15 s at 60°C with a subsequent extension at 68°C for 20 s [39]. To confirm that the primer pair produced only a single specific product, a melting curve was added to the end of the qPCR assay. Cell abundance for field samples was calculated by plotting against a standard curve constructed from DNA extracted from known cell concertation of a *G. polynesiensis* culture (CG14). All data was analysed using BIORAD CFX Manager Software.

### Chemical extraction of *Gambierdiscus* and fish tissue samples for CTX analyses

Microalgal cell pellets were extracted as per the procedure in [40]. Briefly, microalgae cultures were harvested in the stationary phase using centrifugation (3,200 × *g* for 10 min at 10°C) followed by a sonication aided (59 Hz for 10 min) double extraction using 90% aq. MeOH (one mL per 200,000 cells). The resulting supernatants were pooled and stored at –20°C for 24–48 hrs to precipitate sparingly-soluble matrix co-extractives and proteins. The extract was then clarified using centrifugation (3,200 × *g* for 10 min at 4°C) and an aliquot was transferred to a 2 mL glass autosampler vial ready for chemical analysis.

After homogenization of the flesh, CTXs were extracted from a 5 g aliquot following the protocol from [39]. The extraction process resulted in three distinct liposoluble fractions (LF): LF70/30, LF90/10, and LF100. As the majority of CTXs are eluted in the LF90/10 [39,41], only this latter fraction was considered to search for the presence of CTXs. The same procedure was applied on 5 g aliquot of homogenised liver or viscera, retrieved from the same fish samples. For *C. striatus,* viscera samples were pooled from specimens collected in the same site to have sufficient tissue for analyses. For *C. glauca,* specimens were analysed whole (flesh and viscera) as the fish were too small to separate out tissue types. For all other fish species, flesh and viscera/liver samples were analysed separately but tissue types were pooled by species (i.e., all flesh samples from individuals of a species were pooled).

### Liquid chromatography-tandem mass spectrometry (LC-MS/MS)

All fish and microalgal extracts were analysed using the LC-MS/MS method described in [40], with the target compounds identified based on multiple-reaction monitoring (MRM) fragment ion ratios and retention time compared to purified reference material. Certified P-CTX reference material was generated and provided by Professor Takeshi Yasumoto at the Japan Food Research Laboratories (Tokyo, Japan) (P-CTX1B 43.3 ± 1.3 ng; P-CTX2 38.4 ± 2.5 ng (52-epi-54-deoxyCTX1B); P-CTX3C 38.5 ± 2.6 ng; P-CTX4 A 55.1 ± 5.2 ng). Data acquisition and processing was performed using MassLynx and TargetLynx software, respectively. For correlative analysis between *Gambierdiscus* abundance measurements using artificial substrate samplers and CTX3B concentrations in *C. striatus* viscera samples using LCMS, the normality of residuals was assessed by running a linear model. Normality was observed. Therefore, Pearson’s Correlation Coefficient tests were applied. Statistical analyses were performed using R Studio software Version 2024.09.0. For all tests, p-values of <0.05 were considered statistically significant.

### Neuroblastoma cell based assay (CBA-N2a)

The neuroblastoma cell-based assay (CBA-N2a) was done using the mouse N2a cell line (CCL-131) purchased from the American Type Culture Collection (Manassas, VA, USA). Analyses were conducted in the absence and presence of a nondestructive treatment of ouabain (O) and veratridine (V), i.e., in OV- and OV+ conditions, respectively, to detect CTX like-activity according to the optimized protocol published by [42]. A total of 36 fish samples were first analyzed using a qualitative screening at the maximum concentrations of dry extract (MCE) that did not induce unspecific cytotoxic effects in neuroblastoma cells (10,000 pg µL^–1^ for LF90/10 fish extracts).

Among fish samples for which CTX-like activity was detected, twelve of them were selected for quantitative analysis with a serial dilution 1:2 of eight concentrations (final concentrations ranging from 74 to 9524 pg of dry extracts per mL) in both conditions of treatments. Standard solutions of CTX3C and CTX1B sourced from ILM (final concentrations ranging from 0.15 to 19.05 pg mL^-1^) were tested in parallel with fish samples. Each concentration of CTX standards and fish samples were tested in triplicate wells in three independent experiments. Next, the composite CTX-like activity of fish sample was compared to the cytotoxic activity of a pure CTX standard (e.g., CTX3C and CTX1B for herbivores/omnivores and carnivores, respectively) to allow determination of CTX content expressed in ng CTX3C or CTX1B eq./g of fresh tissue [42].

## Results

### Cook Islands field sampling

Six sites were selected for sampling around Rarotonga (S1 Table, Fig 1A) that contained suitable habitat, including for deployment of artificial substrate samplers, and the presence of macroalgal beds. Sites had different dominant macroalgal species, including *Cladophora* sp., *Halimeda* sp.*, Jania* sp.*, Padina* sp., and *Turbinaria ornate* (S1 Table). *Padina* sp. was the most common species and present at all sites except for Tikioki on the south-eastern coast. Baseline data collected over several years (2007–2011) indicated that the south-eastern sites (Muri, Tikioki and Titikaveka) had lower water quality than other sites with a high number of occasions with unacceptable nutrient concentrations (Fig 1A).

### Dinoflagellate diversity and distribution

*Gambierdiscus* was detected at all sites using metabarcoding and was one of the dominant dinoflagellate genera present (Fig 1A). The number of reads identified as *Gambierdiscus* were lowest at the south-eastern sites (Tikioki and Titikaveka). Eight species of *Gambierdiscus* (*G. australe*s, *G. caribaeus*, *G. cheloniae*, *G. honu, G. pacificus, G. polynesiensis* and *G. toxicus*) and one species of *Fukuyoa* (*F. yasumotoi*) were detected (Fig 1A, S1 Fig). *Gambierdiscus polynesiensis* reads were relatively low at all sites except Tikioki. Other potentially toxic dinoflagellate genera detected included *Alexandrium (A. andersonii, A. insuetum), Coolia (C. canariensis), Ostreopsis (O. lenticularis)* and *Prorocentrum (P. emarginatum, P. lima, P. micans, P. sculptile)* (S3 Table).

Dinoflagellate species diversity was similar at all sites except at Muri, where diversity was significantly lower than all other sites (one-way ANOVA with Tukey HSD post-hoc test, p < 0.01) (Fig 1B). ANOSIM comparison of sites (*R* = 0.66, *P* = 0.0001) indicated that dinoflagellate community structure was statistically different between sites. The dinoflagellate community at Muri was the most distinct from all other sites (Fig 1A), with a high proportion of reads identified as *Gambierdiscus* spp. and almost totally dominated by *G. carpenteri*. The rest of the sites clustered together, although the southern sites, Tikioki and Titikaveka, were most similar to each other while the western sites (Papua, Betela and Nikao) also clustered more strongly. No difference in dinoflagellate community structure was detected using the two collection methods and species presence/absence: artificial substrate and macroalgae (Fig 1B; ANOSIM: *R* = −0.05, *P* = 0.75).

### Detection of ciguatoxins in fish samples and *Gambierdiscus* strains

**Fig 2:**
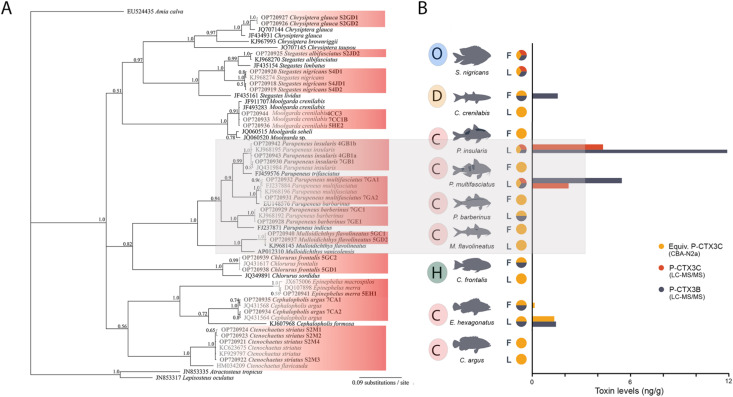
Fish phylogenetic data and toxin data. A. Phylogeny of commonly caught reef fish collected around Rarotonga (in bold) based on an alignment of cytochrome C oxidase I (COI) sequences using Bayesian analysis. Values at nodes represent Bayesian Posterior Probability support. B. Ciguatoxins (CTXs) in flesh (F) and liver (L) samples. Data presented are from liquid chromatography with tandem mass spectrometry (LC-MS/MS; CTX3B (grey) and CTX3C (red)) and neuroblastoma cell-based assay (CBA-N2a; CTX3C equivalents (yellow)) analyses. Fish are categorised depending on the diets as carnivorous (C), herbivorous (H), detritivorous (D) or omnivorous (O). Grey box highlights carnivorous members of the family Mullidae containing taxa with the two highest levels of CTXs measured. Also see S2 Table.

The taxonomic identification of all fish species collected in this study were confirmed using DNA sequencing of the COI gene (Fig 2A3). Nine fish species were opportunistically sampled to determine the extent of CTX presence in a range of fish species around Rarotonga (Fig 2B, S3 Table). CTXs were detected in most fish samples (liver or flesh) using either the CBA-N2a or LC-MS/MS. There was some variation between the two methods with CTX-like activity detected in some of the samples by CBA-N2a only. Levels were quantifiable with LC-MS/MS in flesh and/or liver for the Doublebar goatfish (*Parupeneus insularis*), Manybar goatfish (*Parupeneus multifasciatus*), Mullet (*Moolgarda crenilabis*), and Hexagon Grouper (*Epinephelus hexagonatus*). Two goatfish species (*P. multifasciatus* and *P. insularis*) had the highest levels of CTX3B and CTX3C in the liver samples but no CTXs were detected in the flesh samples (Fig 2B). Conversely, CTX-like activity or CTXs were detected only in the flesh but not in the liver for *Chlorurus frontalis* using CBA-N2a and LC-MS/MS respectively (S3 Table).

**Fig 3:**
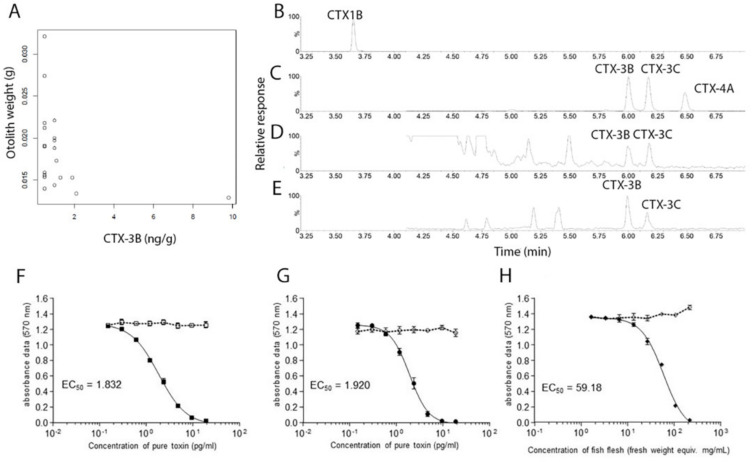
Fish age and toxicity data. A. Otolith weight (proxy for fish size and age) as compared to Ciguatoxin (CTX) concentration, as measured using liquid chromatography with tandem mass spectrometry (LC-MS/MS; CTX3B data shown) results for flesh samples from *Ctenochaetus striatus* collected from Rarotonga, Cook Islands. B. Total ion chromatograms of standards CTX1B, C. CTX3B, CTX3C and CTX4A, and of D. *C. striatus* flesh and E. *C. striatus* viscera. F. Dose-response curves of neuroblastoma N2a cells in OV- (broken line) and OV+ (full line) conditions exposed to reference standard G. CTX3C (a) and H. CTX1B and LF90/10 dry extracts of *Ctenochaetus striatus* flesh (site Tikioki-1). Absorbance data of each concentration represent the mean ± SD of triplicate wells run in one experiment. Sample dry extract concentrations have been transformed in mg fresh weight equiv./mL to facilitate comparison with reference standard at effective concentration showing 50% toxicity.

Using CBA-N2a screening, CTX-like activity was detected in *C. striatus* specimens from all sites (Fig 3). Weight was used as a proxy for age. Fish age versus CTX concentration for flesh samples from *C. striatus* samples was correlated (S2 Fig). This was performed to ensure that any spatial relationships were not confounded by differences in the size/age of fish among sites and to determine any relationship between the age of fish and toxin concentrations. There was no correlation between CTX levels in the viscera of *C. striatus* and the age of the fish, as calculated by otolith weight (Fig 3A).

**Fig 4:**
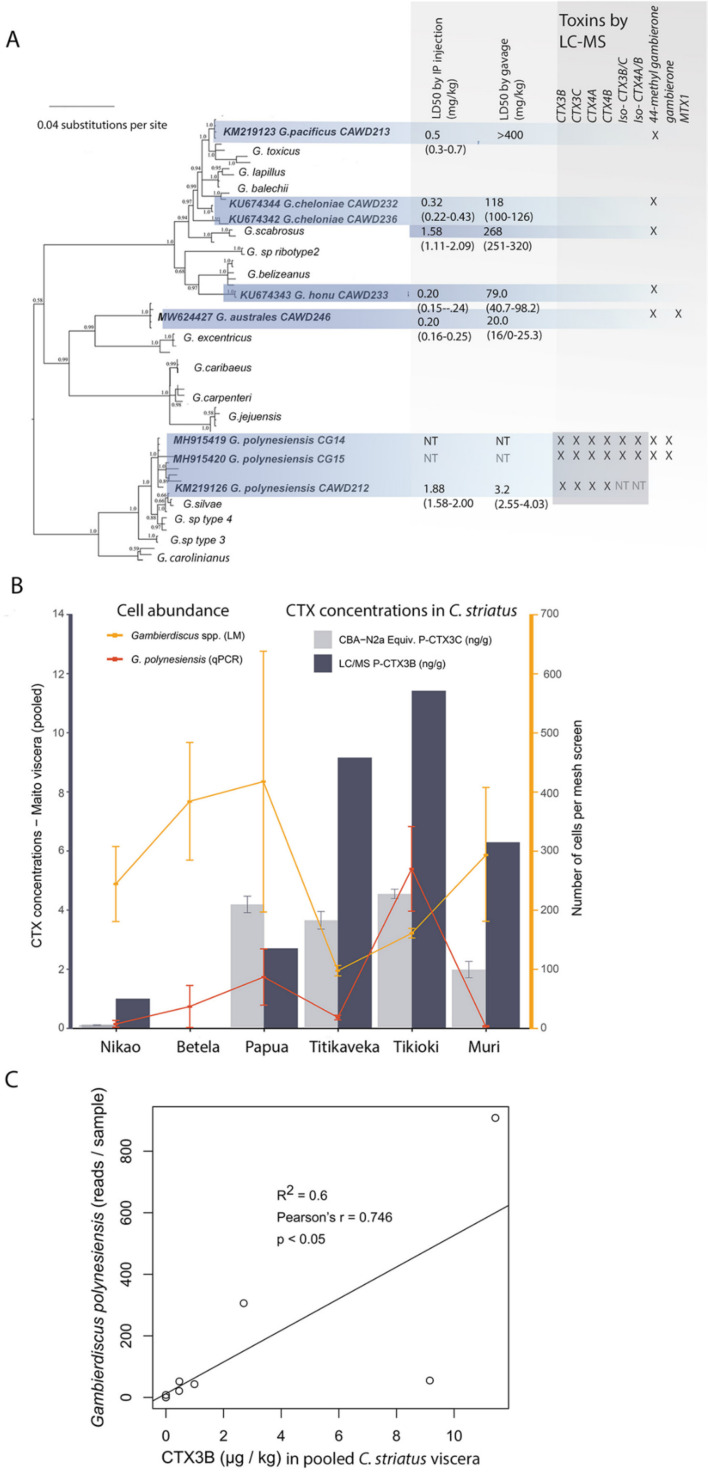
*Gambierdiscus* diversity and toxicity data. A. Phylogeny of *Gambierdiscus* isolates collected from Rarotonga, Cook Islands based on partial large subunit ribosomal DNA (LSU rDNA) sequences using Bayesian analysis. Values at nodes represent Bayesian Posterior Probability support. Characterized toxins using liquid chromatography–mass spectrometry (LC-MS/MS) and acute toxicities by intraperitoneal (i.p.) injection and by gavage (mouse bioassay) are shown. Toxin results presented in 4A are adapted from [14,40,43–45]. B. Ciguatoxin (CTX) results for viscera samples from *Ctenochaetus striatus* at sites in Rarotonga (Betela = no data) using both (LC-MS/MS; only CTX3B data shown) and the neuroblastoma cell-based assay (CBA-N2a; CTX3C equivalents) compared with qPCR results for *G. polynesiensis* and cell counts for all *Gambierdiscus* cells using light microscopy from artificial substrate samplers. C. Correlation of Ciguatoxin (CTX) results for viscera samples from *C. striatus* (LC-MS/MS; CTX3B data) and *G. polynesiensis* metabarcoding read abundance from artificial substrate samplers at sites in Rarotonga. Also see S1 and S2 Figs.

Eight strains representing five species of *Gambierdiscus* were established, including *G. australe*s, *G. cheloniae*, *G. honu, G. pacificus,* and *G. polynesiensis* (Fig 4A). All strains produced 44-methylgambierone (44-MG), while only *G. australes* produced maitotoxin-1 (MTX1) and only *G. polynesiensis* produced known Pacific CTXs (CTX3B, -3C, -4A, -4B, iso-CTX3B/C, iso-CTX4A/B, and gambierone) (Fig 4A). Toxin results presented in Fig 4A are adapted from [14,40,43–45].

Fish collected from Tikioki showed 100% of the specimens (5/5) classified as positive (S4 Table). At the Papua site 60% of specimens (3/5) classified as suspect, although the CTX content of the pooled viscera tissues is among the highest values, especially for Tikioki site (S4 Table). Using LC-MS/MS, only two Pacific CTX analogues were detected in flesh or liver samples, i.e., CTX3B and CTX3C (S4 Table). The highest concentrations of CTXs as determined by LC-MS/MS in *C. striatus* were detected in fish viscera collected in decreasing order from Tikioki, Titikaveka, Muri, and Papua (Fig 4B, S4 Table). No fish could be collected from Betela so toxin data is not available for this site. The LC-MS/MS detected higher levels of CTXs, compared to the CBA-N2a, in seven flesh and three viscera *C. striatus* tissues (Fig 4B, S4 Table). However, LC-MS/MS also gave lower CTX levels in one viscera sample (Site Papua) and was not able to detect or quantify CTXs in four flesh tissues samples that were quantified by CBA-N2a (Site Muri, Site Tikioki, Site Papua, and Site Nikao). No CTX-like activity was detected in the *C. glauca* samples, except for one pooled sample collected from Tikioki that was classified suspect (positive but below the limit of detection) from CBA-N2a screening (S5 Table).

### Toxins correlation with *Gambierdiscus* abundance

The qPCR assay for *G. polynesiensis* had high efficiency (99.9%) and a lower limit of detection of 0.1 cells per PCR assay which converted to five cells per artificial substrate sampler (S4 Fig). Environmental samples run undiluted and diluted by 1:10 showed no evidence of inhibition (i.e., cycle threshold values were approximately 3.3 cycles apart). The highest concentrations of *G. polynesiensis* cells per artificial substrate sampler using qPCR analyses was at Tikioki (Fig 4B). This was in line with the highest levels of CTX in *C. striatus* viscera samples from the same site using both CBA-N2a and LC-MS/MS. All other sites had low levels of *G. polynesiensis* cells detected by qPCR, including at Muri and Titikaveka, yet there were high levels of CTXs in *C. striatus* liver at both these sites – in close proximately to Tikioki. *Gambierdiscus polynesisensis* qPCR and metabarcoding results were highly aligned (*r* = 0.995, *p* < 0.05) and so additional metabarcoding and *C. striatus* samples collected in 2022 were merged with 2014 data to establish a correlation between *G. polynesisensis* abundance and CTX3B concentrations in fish. A positive correlation between metabarcoding read abundance for *G. polynesiensis* and CTX3B levels in viscera samples from *C. striatus* collected around Rarotonga (2014 and 2022 samples combined) was established (*r* = 0.746, *p* = 0.05) (Fig 4C). *Gambierdiscus* cells were detected at all sites via microscopy. In contrast to the metabarcoding results and CTX concentrations, the sites Tikioki and Titikaveka had the lowest *Gambierdiscus* spp. cell concentrations, although variation between samples was high (Fig 4B). There was a non-significant negative correlation between the microscopy cells concentrations (i.e., all *Gambiediscus* spp.) and CTX levels in the fish samples (*r* = −0.69, *p* = 0.20).

## Discussion

CP is one of the world’s most under-recognised and difficult to manage food safety risks, despite affecting tens of thousands of people annually throughout much of the tropical and subtropical world [3]. The Cook Islands has one of the world’s highest rates of CP, with well documented ecological, economic, and social impacts [29]. Despite decades of research, the correlation between the abundance of confirmed *Gambierdiscus* CTX producers in relation to specific CTX concentrations and analogues in fishes at specific locations has been difficult or impossible to predict, preventing the development and implementation of effective monitoring programmes. Some studies have detected the *Gambierdiscus* genus or individual *Gambierdiscus* species using molecular methods, but as the toxicities of isolates were not confirmed, they were not able to link the presence of *Gambierdiscus* (to the species level) to specific CTX analogues (using the gold standard method of LC-MS/MS) in fish species [e.g., 15,39,41,46,47,48], while other studies isolated strains of *Gambierdiscus* and confirmed they produced CTXs, but did not show localised food web related uptake [e.g., 50]. Some studies have shown similar relationships using cell-based assays or invertebrate species [15,41,47–49]. In this study, using a combination of approaches, we were able to identify CTX analogues in a sentinel omnivorous fish with a localised home range (maito/striated surgeonfish; *Ctenochaetus striatus*) and coupled these data with metabarcoding of the epiphytic eukaryotic microbial community (identification to the species level). We also characterised toxin production from several dinoflagellate strains, used taxon-specific qPCR, DNA metabarcoding and applied LC-MS/MS and bioassay methods of toxin identification, to establish links between CTX microbial producers and CTX prevalence in the marine food web.

Of the novel strains of *Gambierdiscus* studied*,* only the isolates of *G. polynesiensis* produced known CTXs (Pacific CTX3B, -3C, -4A, -4B, iso-CTX3B/C, and iso-4A/B). Using multiple different bioassays, many *Gambierdiscus* and some *Fukuyoa* species have demonstrated CTX-like activity, although the identity of the toxin(s) responsible was not described [13,15,18,20,50,51]. Previous research has suggested that *G. australes* and *G. pacificus* may be important species in contributing to CP in the Pacific Ocean region [1], with both species having been shown (on multiple occasions) to have CTX-like activity using bioassays, including the receptor binding assay, mouse bioassay, and the CBA-N2a [13,15,20,50,51]. In those studies, *G. australes* was suggested to produce up to 0.6 pg CTX3C equivalence cell^-1^, which is within the range of values of CTX cell^-1^ production of examined strains of *G. polynesiensis* [13,14,16,17,52]. Isolates of *G. pacificus* and *G. australes* from this field study did not produce known CTXs as determined by LC-MS/MS, and no relationship between *G. pacificus* and *G. australes* presence and CTXs in *C. striatus* were found. This highlights the important role *G. polynesiensis* plays in determining CP risk in the South Pacific region.

Coral reef microbiomes, including epiphytic communities on macroalgae, show significant spatial patchiness but temporal stability in composition and diversity over long time periods (i.e., months or longer) [53]. Hence, spatial sampling was a focus of this study. Alpha diversity was significantly lower only at Muri. The lagoon at Muri is a significant tourist attraction and the area contains the largest density of tourist accommodation on the island. Urbanisation, farming and tourism surrounding Muri lagoon may have contributed to poor water quality and nuisance macroalgal blooms that occur in this part of the lagoon [54]. Poor water quality and presence different macroalgal habitat associated with this may be responsible for lower α diversity levels, with the *Gambierdiscus* community dominated by *G. carpenteri* a species known to be more highly tolerant of variable water conditions [9]. The use of artificial substrates for epiphytic biological sample collection has been found to provide a more standardised sampling approach than measuring abundance on macroalgal surfaces [55]. In our study, dinoflagellate species presence/absence data was equivalent across both artificial substrate and macroalgae sampling methods, as sites clustered regardless of collection method, suggesting that sampling approaches can be tailored based on availability and preference.

For many years, *Gambierdiscus* was considered monotypic, and few species were known until the past decade [56]. Diversity ‘hotspots’ assemblages of five-to-six *Gambierdiscus/Fukuyoa* species have been identified recently in the Pacific (French Polynesia [57], Japan [51], Australia [58–61] and Kiribati [62]) as well as the Atlantic Ocean and Caribbean (Gulf of Mexico [63], Cuba [46], and the Canary Islands [64]). In this study, eight species of *Gambierdiscus* and one *Fukuyoa* species were detected, the highest reported in any single study, in this case from one small island (ca. 11 km wide). A further species, *G. lapillus* was also detected in Rarotonga recently [65]. It seems unlikely that Rarotonga has unusually diverse epiphytic microbial eukaryotic assemblages compared to other Pacific tropical coral reefs but rather that *Gambierdiscus* diversity remains underestimated worldwide. Deep sequencing of multiple samples, and on-going sampling from the same location, may be necessary to uncover *Gambierdiscus* taxa present in the microbial ‘rare biosphere’ [66].

To date, metabarcoding of marine microbial eukaryotes has focused on planktonic communities, generally using V4 or V9 regions of the 18S rDNA [67,68]. Relatively short and conserved gene regions may not always be able to distinguish at the species-level [24], while our primer pair targeting a region of the LSU fully resolved dinoflagellates at the species level [12]. As CTX production by different *Gambierdiscus* appear to vary by several orders of magnitude (up to >10 pg cell^-1^) [13,52], very rare taxa may nevertheless be crucial to understanding CTX transmission through a local food web [16,52,49]. In this sense, it may not be necessary for a *Gambierdiscus* taxon to be present in unusually high abundances (i.e., ‘blooming’) to contribute to CP occurrence.

While most sites had dinoflagellate assemblages heavily dominated by *Gambierdiscus/Fukuyoa*, only sites with high abundance of *G. polynesiensis* aligned with CTX3B concentrations in *C. striatus* rather than total *Gambierdiscus* spp. abundance*,* and this was unrelated to fish age or size. Locations with higher cell abundance of *G. polynesiensis* were positively correlated with higher CTX levels in the viscera of *C. striatus*. The highest concentrations of *G. polynesiensis* cells per artificial substrate using qPCR analyses was at Tikioki. This was in line with the highest levels of CTX in *C. striatus* viscera samples from the same site using both CBA-N2a and LC-MS/MS. Field studies in French Polynesia have indicated local influences of *Gambierdiscus* spp. abundance on the CTX toxicity of fish or marine invertebrates [15,39,41,48] but not previously tested it using a standardised fish as a sentinel species. Given high variability in CTXs in commonly caught reef fish, a better indicator of site-specific risk over time is a sentinel species with a small home range that can be compared in a standardised way. *Ctenochaetus striatus* was ranked in the top three fishes that cause CP in the Cook Islands [29]. It has a wide distribution in the Indian and Pacific Oceans, a small feeding home range, is a detrital aggregate feeder, and may also act as a secondary herbivore [69]. Our results demonstrate that *C. striatus* is an appropriate sentinel fish species for CP the Pacific region, as it was indicative of localised CTX accumulation, independent of individual fish age or size, suggesting that age or size data would not be required.

CTX presence was ubiquitous in fish species collected opportunistically during this study, with detections from all locations which highlights the risk of CP from seafood collected from the lagoon. None of the fish contained fish CTX metabolites (e.g., CTX1B) that are known to be highly toxic and the main cause of CP in humans, however, the analogues of CTXs produced by *G. polynesiensis* such as CTX4A, CTX4B, and CTX3C, are also toxic and have been found in herbivorous fish species [47,70], as was the case in the present study. Bioconversion into different CTX analogues and the trophic transfer from herbivorous to carnivorous fish is therefore not a prerequisite for CTX accumulation, as fish can become ciguatoxic without trophic transfer from herbivorous to carnivorous fish, or biomagnification of toxins [70]. The prevalence of microalgal CTX analogues in almost all fish tested from around Rarotonga may represent a high risk of CTXs in fish at higher trophic levels in the region, although this remains to be investigated.

## Conclusion

Multiple long-term drivers can increase the abundance of *Gambierdiscus* and CTXs, and therefore the risk of CP, including ocean warming, increased nutrient loading in lagoons, coral bleaching, reef disturbance, and changes to ocean currents [5,6,8–10,71]. CP therefore requires a ‘One Health’ approach to mitigate its impacts. The field of CP research has established important principles of CTX production and transmission in aquatic food webs. Despite these advances, particular *Gambierdiscus* species/populations cannot be linked to CTXs in given reef fish populations, and the exact source of CTXs in an area often remains unknown. This has inhibited the establishment of a basis for comparative CP risk assessment. Under-sampling of *Gambierdiscus* spp. as part of the ‘rare biosphere’ may have occurred, and many or most *Gambierdiscus* have variably been experimentally shown to produce some CTX-like activity in cell-based assays, leaving the source of local CTXs often unclear. Here, using a multifaceted approach, we were able to identify CTXs in a sentinel fish with a small home range, and associate it with one comparatively rare *Gambierdiscus* species, which produced the same CTX analogues using LC-MS/MS analyses. As *C. striatus* appears to be an excellent, widely applicable model taxon. A single microbial taxon (in this case, *G. polynesiensis*) can be monitored relatively inexpensively using methods such as on-site qPCR, and may require only infrequent (i.e., six monthly) monitoring to detect changes in relatively stable epiphytic communities [53]. Due to differences of several orders of magnitude in microbial CTX production, a reference microbial baseline including the rare biosphere appears to be an important initial step. If this approach were coupled with chemical CTX measurement in a sentinel fish where practical, it may provide a standardised marker for toxicity over spatial and long-term temporal scales. Given that long term CP risk is expected to expand locally in relation to climate change increasing the frequency and severity of tropical cyclones, marine heat waves, and high likelihood of disturbances to coral reefs, such detection may be important in protecting public health across SIDs and other countries from this neglected tropical disease.

## Supporting information

S1 Table Sampling site information for the six locations around Rarotonga (Cook Islands) including dominant macroalgal species sampled for dinoflagellate communities. n = number of samples collected.
(DOCX)

S2 Table Fish dietary information for species collected for this study. Information obtained from Fishbase (www.fishbase.org).
(DOCX)

S3 Table Metabarcoding read numbers for the large subunit ribosomal RNA gene, V4 region from samples collected at each site around Rarotonga (Cook Islands) in November 2014. (DOCX)

S4 Table Ciguatoxin (CTX) results for viscera and flesh samples from *Ctenochaetus striatus* collected from each of the sites around Rarotonga, Cook Islands for both liquid chromatography with tandem mass spectrometry (LC-MS/MS; CTX3B and CTX3C data shown) and the neuroblastoma cell-based assay (CBA-N2a) with screening results of CTX-like activity ranged in three categories (negative, suspect, positive) and quantitative results in ng CTX3C equivalents/g of tissue. NT: not tested for quantification; ND: not detected.
(DOCX)

S5 Table Ciguatoxin (CTX) results for flesh and liver samples from fish species collected from each of the sites around Rarotonga, Cook Islands for both liquid chromatography with tandem mass spectrometry (LC-MS/MS; CTX3B and CTX3C data shown) and the neuroblastoma cell-based assay (CBA-N2a; CTX3C equivalents). ND = not detected; Pacific ciguatoxins = CTX1B, CTX3C; CTX3B. ND = not detected, NT = not tested for quantification.
(DOCX)

S6 Table Ciguatoxin (CTX) results for *Chrysiptera glauca* collected from each of the sites around Rarotonga, Cook Islands for both liquid chromatography with tandem mass spectrometry (LC-MS/MS; CTX3B and CTX3C data shown) and using the neuroblastoma cell-based assay (CBA-N2a) with screening results of CTX-like activity ranged in three categories (negative, suspect, positive). Fish samples were homogenized whole and five fish were pooled from each location. ND = not detected.
(DOCX)

S1 Fig Phylogenetic analysis of large subunit ribosomal RNA (LSU) sequences obtained from the high-throughput sequencing (HTS) metabarcoding from sampling sites around Rarotonga (Cook Islands). Sequences in bold represent the consensus sequence from all reads of each taxonomic assignment. Values at nodes represent Bayesian posterior probability support. Scale bar is substitutions per site.
(DOCX)

S2 Fig **A. Linear regression analysis showing the relationship between the length of** *Ctenochaetus striatus* **and the weight of its otolith.** B. Linear regression analysis showing the relationship between the length of *C. striatus* and the age estimate of its otolith.
(DOCX)

S3 Fig Phylogenetic analyses using the large subunit ribosomal RNA (LSU) (D1-D3 region) sequences from *Gambierdiscus* strains isolated during this study from sampling sites around Rarotonga (Cook Islands) using Bayesian analyses. Values at nodes represent Bayesian posterior probability support. Scale bar is substitutions per site.
(DOCX)

S4 Fig Standard curve for the *Gambierdiscus polynesiensis* real-time PCR assay constructed with 10-fold serial dilutions of genomic DNA extracts from available *G. polynesiensis* culture (CG14). (DOCX)
