# Correction: Risk of ciguatoxins is shaped by *Gambierdiscus* community structure

**DOI:** 10.1371/journal.pone.0345647

**Published:** 2026-03-20

**Authors:** Kirsty F. Smith, Lesley L. Rhodes, Belinda Curley, Arjun Verma, Gurjeet Kohli, David Tim Harwood, James Sam Murray, Jérôme Viallon, Hélène Taiana Darius, Mireille Chinain, Teina Rongo, June Hosking, Phoebe A. Argyle, Jacqui Stuart, Shauna A. Murray

There are errors in the author affiliations. The correct affiliations are as follows:

Kirsty F. Smith^1^, Lesley L. Rhodes^1^, Belinda Curley^2^, Arjun Verma^3^, Gurjeet Kohli^3^, D. Tim Harwood^1^, J. Sam Murray^1^, Jérôme Viallon^4^, Hélène Taiana Darius^4^, Mireille Chinain^4^, Teina Rongo^5^, June Hosking^6^, Phoebe A. Argyle^1^, Jacqui Stuart^1^, Shauna A. Murray^3^

**1** Cawthron Institute, Nelson, New Zealand, **2** NSW Department of Primary Industries, Port Stephens, New South Wales, Australia, **3** University of Technology Sydney, School of Life Sciences, Sydney, New South Wales, Australia, **4** Laboratory of Marine Biotoxins, Institut Louis Malardé, UMR-241 EIO (Ifremer, ILM, IRD, UPF), Tahiti, French Polynesia, **5** Kōrero O Te `Ōrau, Rarotonga, Cook Islands, **6** Mauke, Cook Islands
