# Supplementary material for: Risk of ciguatoxins is shaped by Gambierdiscus community structure
Source: PLoS One. 2026 Jan 29;21(1):e0341899. doi: 10.1371/journal.pone.0341899 (PMC12854468; doi:10.1371/journal.pone.0341899)
Supplement: S1 Table — n = number of samples collected. (DOCX) [file pone.0341899.s001.docx]

**Supplementary Table 1.** Sampling site information for the six locations around Rarotonga (Cook Islands) including dominant macroalgal species sampled for dinoflagellate communities. n = number of samples collected.

| **Site name** | **Location co-ordinates** | **Dominant macroalgal species** |
| --- | --- | --- |
| Muri | 21°15'32'' S, 159°43' 71'' W | *Padina* sp. (n = 3), *Cladophora* sp. (n = 3) |
| Tikioki | 21°16'24'' S, 159°44' 53'' W | *Halimeda* sp. (n = 6) |
| Titikaveka | 21°16'50'' S, 159°46' 50'' W | *Padina* sp. (n = 3), *Cladophora* sp. (n = 3) |
| Papua | 21°15' 75'' S, 159°47' 84'' W | *Padina* sp. (n = 3), *Jania* sp. (n = 3) |
| Betela | 21°14' 96'' S, 159°49' 38'' W | *Padina* sp. (n = 3), *Turbinaria ornata* (n = 3) |
| Nikao | 21°12' 40'' S, 159°49' 33'' W | *Padina* sp. (n = 5), *Turbinaria ornata* (n = 1) |

**Supplementary Table 2.**  Fish dietary information for species collected for this study. Information obtained from Fishbase ([www.fishbase.org](http://www.fishbase.org)).

| **Cook Islan/Common name** | **Species name** | **Family** | **Trophic group** | **Diet (Fishbase.org)** |
| --- | --- | --- | --- | --- |
| U'u'/Parrotfish | *Chlorurus frontalis* | Scaridae | Herbivore | Benthic algae |
| Katoti/Grey damselfish | *Chrysiptera glauca* | Pomacentridae | Omnivore/ herbivore | Feed mainly on benthic algae. |
| Katoti/Dusky damselfish | *Stegastes nigricans* | Pomacentridae | Omnivore | Feed on algae, gastropods, sponges, and copepods. Territorial, maintain and 'weed' filamentous algae patches growing on dead coral |
| Maito/Striated surgeonfish | *Ctenochaetus striatus* | Acanthuridae | Omnivore/ Detritivore | Feeds on surface film of blue-green algae and diatoms (making this species a key link in the ciguatera food chain) as well as on various small invertebrates. |
| Kanae/Mullet | *Crenimugil crenilabis* | Mugilidae | Detritivore | Feed on detritus containing algae and microscopic animals, by scooping up the upper layer of sand or mud and filtering through the gills |
| Pātuki roi/Peacock grouper | *Cephalopholis argus* | Serranidae | Carnivore | Adults feed mainly on fish |
| Pātuki paru/Hexagon Grouper | *Epinephelus hexagonatus* | Serranidae | Carnivore | Feeds mainly on fishes and crustaceans |
| Ka‘uru/Manybar goatfish | *Parupeneus multifasciatus* | Mullidae | Carnivore | Feeds primarily on small crabs and shrimps during the day also demersal fish eggs, mollusks, and foraminiferans |
| Ka‘uru/Doublebar goatfish | *Parupeneus insularis* | Mullidae | Carnivore | Stomach contents of 12 specimens consist of 44% by volume of crabs; followed by shrimps, octopuses, mantis shrimps, amphipods and other crustaceans, fishes, and polychaetes |
| Ka‘uru/Dash-and-dot goatfish | *Parupeneus barberinus* | Mullidae | Carnivore | 19 adult specimens for food-habit study, the prey in order by volume in the stomachs: crabs (portunid, anomuran, xanthid, and raninid), worms (mainly polychaetes but also sipunculids and unidentified), small bivalve molluscs, brachiopods, shrimps, small gastropods, isopods, amphipods, foraminifera, and a small unidentified eel |
| Vete/Yellowstripe goatfish | *Mulloidichthys flavolineatus* | Mullidae | Carnivore | Feed on crustaceans, mollusks, worms, heart urchins and foraminiferans. |

**Supplementary Table 3.** Metabarcoding read numbers for the large subunit ribosomal RNA gene, V4 region from samples collected at each site around Rarotonga (Cook Islands) in November 2014.

|  | **Number of reads** | | | | | |
| --- | --- | --- | --- | --- | --- | --- |
|  | **Muri** | **Titioki** | **Titikaveka** | **Papua** | **Betela** | **Nikao** |
| *Phalacroma rapa* | 24 | 21 | 95 | 127 | 30 | 27 |
| *Gambierdiscus australes* | 1580 | 4960 | 1651 | 15362 | 9331 | 17334 |
| *Gambierdiscus honu* | 1409 | 288 | 271 | 3629 | 487 | 1714 |
| *Gambierdiscus caribaeus* | 11 | 16 | 0 | 0 | 1530 | 0 |
| *Gambierdiscus carpenteri* | 52645 | 6278 | 4764 | 8791 | 0 | 2599 |
| *Gambierdiscus pacificus* | 1519 | 958 | 733 | 2072 | 1912 | 2486 |
| *Gambierdiscus polynesiensis* | 4 | 908 | 55 | 306 | 8 | 43 |
| *Gambierdiscus toxicus* | 1 | 0 | 0 | 35 | 0 | 20 |
| *Fukuyoa yasumotoi* | 3 | 0 | 0 | 96 | 0 | 130 |
| *Gambierdiscus cheloniae* | 1725 | 242 | 218 | 981 | 161 | 218 |
| *Alexandrium andersonii* | 63 | 308 | 1127 | 9 | 115 | 0 |
| *Alexandrium insuetum* | 0 | 42 | 20 | 9 | 0 | 51 |
| *Gonyaulax* sp. | 16 | 38 | 18 | 0 | 0 | 0 |
| Gonyaulacaceae unclassified | 1 | 21 | 34 | 0 | 53 | 106 |
| Gonyaulacales unclassified | 126 | 46 | 17 | 107 | 0 | 0 |
| *Amphidinium carterae* | 165 | 421 | 281 | 361 | 98 | 700 |
| *Amphidinium gibbosum* | 0 | 188 | 0 | 126 | 0 | 0 |
| *Amphidinium massartii* | 1216 | 1507 | 1523 | 1368 | 333 | 2256 |
| *Amphidinium* sp. | 0 | 21 | 50 | 0 | 0 | 18 |
| *Amphidinium steinii* | 0 | 47 | 39 | 24 | 19 | 89 |
| *Amphidinium thermaeum* | 478 | 1534 | 1007 | 2573 | 125 | 2320 |
| *Amphidinium trulla* | 15 | 0 | 26 | 0 | 14 | 48 |
| *Cochlodinium polykrikoides* | 56 | 139 | 301 | 10 | 38 | 0 |
| *Gymnodinium dorsalisulcum* | 1734 | 365 | 717 | 355 | 649 | 1237 |
| *Gyrodinium dominans* | 0 | 0 | 46 | 127 | 83 | 16 |
| *Lepidodinium chlorophorum* | 0 | 0 | 0 | 0 | 0 | 12 |
| Gymnodiniaceae unclassified | 1011 | 1219 | 1334 | 0 | 323 | 3453 |
| Gymnodiniales unclassified | 37 | 0 | 22 | 1491 | 0 | 0 |
| *Noctiluca scintillans* | 260 | 110 | 303 | 147 | 122 | 251 |
| *Heterocapsa triquetra* | 0 | 80 | 341 | 0 | 0 | 0 |
| Heterocapsaceae unclassified | 0 | 0 | 12 | 0 | 0 | 0 |
| *Coolia canariensis* | 10 | 22 | 9 | 38 | 14 | 20 |
| *Ostreopsis lenticularis* | 5080 | 3681 | 5292 | 3892 | 2795 | 2163 |
| Ostreopsidaceae unclassified | 2617 | 10967 | 20183 | 1586 | 3535 | 2162 |
| *Durinskia baltica* | 177 | 340 | 544 | 47 | 196 | 1123 |
| Peridiniaceae unclassified | 0 | 0 | 0 | 0 | 0 | 11 |
| Peridiniales unclassified | 24 | 31 | 97 | 22 | 24 | 39 |
| *Prorocentrum emarginatum* | 75 | 214 | 72 | 476 | 52 | 292 |
| *Prorocentrum lima* | 37 | 0 | 32 | 22 | 0 | 50 |
| *Prorocentrum micans* | 111 | 684 | 2001 | 707 | 1602 | 473 |
| *Prorocentrum sculptile* | 12 | 19 | 18 | 125 | 73 | 378 |
| *Prorocentrum* sp. | 28 | 29 | 86 | 25 | 62 | 56 |
| Prorocentrales unclassified | 0 | 721 | 0 | 815 | 1399 | 0 |
| Prorocentraceae unclassified | 498 | 0 | 1318 | 0 | 0 | 869 |
| *Biecheleria cincta* | 0 | 0 | 12 | 0 | 0 | 0 |
| *Polarella* sp. | 51 | 398 | 373 | 0 | 32 | 74 |
| *Symbiodinium* sp. | 13 | 18 | 34 | 0 | 0 | 0 |
| *Symbiodinium* sp. type A | 0 | 45 | 74 | 11 | 13 | 76 |
| Symbiodiniaceae unclassified | 122 | 458 | 606 | 258 | 218 | 723 |
| Suessiales unclassified | 95 | 238 | 144 | 17 | 23 | 69 |

**Supplementary Table 4.** Ciguatoxin (CTX) results for viscera and flesh samples from *Ctenochaetus striatus* collected from each of the sites around Rarotonga, Cook Islands for both liquid chromatography with tandem mass spectrometry (LC-MS/MS; CTX3B and CTX3C data shown) and the neuroblastoma cell-based assay (CBA-N2a) with screening results of CTX-like activity ranged in three categories (negative, suspect, positive) and quantitative results in ng CTX3C equivalents/g of tissue. NT: not tested for quantification; ND: not detected.

|  |  |  |  | **CBA-N2a** | | **LC-MS** | |
| --- | --- | --- | --- | --- | --- | --- | --- |
| **Cook Islands/Common name** | **Scientific name** | **Matrix** | **Replicate** | **CTX-like activity** | **CTX content  (ng CTX3C eq./g)** | **CTX3B (ng/g)** | **CTX3C (ng/g)** |
| Maito/Striated surgeonfish | *Ctenochaetus striatus* | Flesh | Site Muri - 1 | positive | 0.58 ± 0.04 | Detected | ND |
|  |  |  | Site Muri - 2 | positive | NT | Detected | ND |
|  |  |  | Site Muri - 3 | positive | NT | 1.92 | ND |
|  |  |  | Site Muri - 4 | suspect | NT | <1 | ND |
|  |  |  | Site Muri - 5 | positive | NT | Detected | ND |
|  |  |  | Site Titioki – 1 | positive | 0.21 ± 0.01 | Detected | ND |
|  |  |  | Site Titioki – 2 | positive | NT | Detected | ND |
|  |  |  | Site Titioki – 3 | positive | NT | 1.12 | ND |
|  |  |  | Site Titioki – 4 | positive | NT | Detected | ND |
|  |  |  | Site Titioki – 5 | positive | NT | Detected | ND |
|  |  |  | Site Titikaveka – 1 | suspect | NT | Detected | ND |
|  |  |  | Site Titikaveka – 2 | positive | 0.19 ± 0.01 | 0.953333333 | ND |
|  |  |  | Site Titikaveka – 3 | positive | NT | 9.8 | 1.5 |
|  |  |  | Site Titikaveka – 4 | positive | NT | 2.146666667 | ND |
|  |  |  | Site Titikaveka – 5 | positive | NT | 1.32 | ND |
|  |  |  | Site Papua - 1 | positive | 0.14± 0.01 | ND | ND |
|  |  |  | Site Papua - 2 | suspect | NT | Detected | ND |
|  |  |  | Site Papua - 3 | suspect | NT | Detected | ND |
|  |  |  | Site Papua - 4 | suspect | NT | Detected | ND |
|  |  |  | Site Papua - 5 | positive | NT | ND | ND |
|  |  |  | Site Nikao - 1 | positive | 0.08 ± 0.01 | ND | ND |
|  |  | Viscera | Site Muri composite | positive | 1.97 ± 0.28 | 6.3 | Detected |
|  |  |  | Site Titioki composite | positive | 4.55 ± 0.16 | 11.43333333 | Detected |
|  |  |  | Site Titikaveka composite | positive | 3.65 ± 0.30 | 9.156666667 | Detected |
|  |  |  | Site Papua composite | positive | 4.19 ± 0.28 | 2.7 | 2.7 |

**Supplementary Table 5.** Ciguatoxin (CTX) results for flesh and liver samples from fish species collected from each of the sites around Rarotonga, Cook Islands for both liquid chromatography with tandem mass spectrometry (LC-MS/MS; CTX3B and CTX3C data shown) and the neuroblastoma cell-based assay (CBA-N2a; CTX3C equivalents). ND = not detected; Pacific ciguatoxins = CTX1B, CTX3C; CTX3B. ND = not detected, NT = not tested for quantification.

|  |  |  | **CBA-N2a** | | **LC-MS/MS** | |
| --- | --- | --- | --- | --- | --- | --- |
| **Cook Islands/Common name** | **Scientific name** | **Matrix** | **CTX-like activity** | **CTX content (ng CTX1B eq./g)** | **CTX3B** | **CTX3C** |
| Pātuki roi/Peacock grouper | *Cephalopholis argus* | Flesh | positive | NT | ND | ND |
|  |  | Liver | positive | NT | ND | ND |
| U'u'/Parrotfish | *Chlororus frontalis* | Flesh | positive | NT | Detected | ND |
|  |  | Liver | suspect | NT | ND | ND |
| Pātuki paru/Hexagon Grouper | *Epinephelus hexagonatus* | Flesh | positive | 0.04 ± 0.00 | Detected | ND |
|  |  | Viscera | positive | 1.29 ± 0.16 | 1.4 | ND |
| Kanae/Mullet | *Crenimugil crenilabis* | Flesh | positive | NT | 1.52 | ND |
|  |  | Liver | positive | NT | ND | ND |
| Ka‘uru/Manybar goatfish | *Parupeneus multifasciatus* | Flesh | suspect | NT | ND | ND |
|  |  | Liver | positive | NT | 5.45 | 2.2 |
| Ka‘uru/Doublebar goatfish | *Parupeneus insularis* | Flesh | suspect | NT | ND | ND |
|  |  | Liver | positive | NT | 11.88 | 4.3 |
| Ka‘uru/Dash-and-dot goatfish | *Parupeneus barberinus* | Flesh | positive | NT | ND | ND |
|  |  | Liver | positive | NT | Detected | ND |
| Vete/Yellowstripe goatfish | *Mulloidichthys flavolineatus* | Flesh | positive | NT | ND | ND |
|  |  | Liver | positive | NT | ND | ND |
| Katoti/Dusky damsel | *Stegastes nigricans* | Flesh | positive | NT | Detected | Detected |
|  |  | Viscera | positive | NT | Detected | Detected |

**Supplementary Table 6.** Ciguatoxin (CTX) results for *Chrysiptera glauca* collected from each of the sites around Rarotonga, Cook Islands for both liquid chromatography with tandem mass spectrometry (LC-MS/MS; CTX3B and CTX3C data shown) and using the neuroblastoma cell-based assay (CBA-N2a) with screening results of CTX-like activity ranged in three categories (negative, suspect, positive). Fish samples were homogenized whole and five fish were pooled from each location. ND = not detected.

|  |  |  |  | **CBA-N2a** | **LC-MS/MS** | |
| --- | --- | --- | --- | --- | --- | --- |
| **Cook Islands/Common name** | **Scientific name** | **Matrix** | **Replicate** | **CTX-like activity** | **CTX3B (ng/g)** | **CTX3C (ng/g)** |
| Katoti/Grey damsel | *Chrysiptera glauca* | whole | Site Muri | Negative | ND | ND |
|  |  |  | Site Titioki | Suspect | ND | ND |
|  |  |  | Site Titikaveka | Negative | ND | ND |
|  |  |  | Site Papua | Negative | ND | ND |
|  |  |  | Betela | Negative | ND | ND |
|  |  |  | Nikao | Negative | ND | ND |
