# Supplementary material for: Risk of ciguatoxins is shaped by Gambierdiscus community structure
Source: PLoS One. 2026 Jan 29;21(1):e0341899. doi: 10.1371/journal.pone.0341899 (PMC12854468; doi:10.1371/journal.pone.0341899)
Supplement: S2 Table — Information obtained from Fishbase (www.fishbase.org). (DOCX) [file pone.0341899.s002.docx]

**Supplementary Table 2.**  Fish dietary information for species collected for this study. Information obtained from Fishbase ([www.fishbase.org](http://www.fishbase.org)).

| **Cook Islan/Common name** | **Species name** | **Family** | **Trophic group** | **Diet (Fishbase.org)** |
| --- | --- | --- | --- | --- |
| U'u'/Parrotfish | *Chlorurus frontalis* | Scaridae | Herbivore | Benthic algae |
| Katoti/Grey damselfish | *Chrysiptera glauca* | Pomacentridae | Omnivore/ herbivore | Feed mainly on benthic algae. |
| Katoti/Dusky damselfish | *Stegastes nigricans* | Pomacentridae | Omnivore | Feed on algae, gastropods, sponges, and copepods. Territorial, maintain and 'weed' filamentous algae patches growing on dead coral |
| Maito/Striated surgeonfish | *Ctenochaetus striatus* | Acanthuridae | Omnivore/ Detritivore | Feeds on surface film of blue-green algae and diatoms (making this species a key link in the ciguatera food chain) as well as on various small invertebrates. |
| Kanae/Mullet | *Crenimugil crenilabis* | Mugilidae | Detritivore | Feed on detritus containing algae and microscopic animals, by scooping up the upper layer of sand or mud and filtering through the gills |
| Pātuki roi/Peacock grouper | *Cephalopholis argus* | Serranidae | Carnivore | Adults feed mainly on fish |
| Pātuki paru/Hexagon Grouper | *Epinephelus hexagonatus* | Serranidae | Carnivore | Feeds mainly on fishes and crustaceans |
| Ka‘uru/Manybar goatfish | *Parupeneus multifasciatus* | Mullidae | Carnivore | Feeds primarily on small crabs and shrimps during the day also demersal fish eggs, mollusks, and foraminiferans |
| Ka‘uru/Doublebar goatfish | *Parupeneus insularis* | Mullidae | Carnivore | Stomach contents of 12 specimens consist of 44% by volume of crabs; followed by shrimps, octopuses, mantis shrimps, amphipods and other crustaceans, fishes, and polychaetes |
| Ka‘uru/Dash-and-dot goatfish | *Parupeneus barberinus* | Mullidae | Carnivore | 19 adult specimens for food-habit study, the prey in order by volume in the stomachs: crabs (portunid, anomuran, xanthid, and raninid), worms (mainly polychaetes but also sipunculids and unidentified), small bivalve molluscs, brachiopods, shrimps, small gastropods, isopods, amphipods, foraminifera, and a small unidentified eel |
| Vete/Yellowstripe goatfish | *Mulloidichthys flavolineatus* | Mullidae | Carnivore | Feed on crustaceans, mollusks, worms, heart urchins and foraminiferans. |
