# Supplementary material for: Risk of ciguatoxins is shaped by Gambierdiscus community structure
Source: PLoS One. 2026 Jan 29;21(1):e0341899. doi: 10.1371/journal.pone.0341899 (PMC12854468; doi:10.1371/journal.pone.0341899)
Supplement: S3 Table — (DOCX) [file pone.0341899.s003.docx]

**Supplementary Table 3.** Metabarcoding read numbers for the large subunit ribosomal RNA gene, V4 region from samples collected at each site around Rarotonga (Cook Islands) in November 2014.

|  | **Number of reads** | | | | | |
| --- | --- | --- | --- | --- | --- | --- |
|  | **Muri** | **Tikioki** | **Titikaveka** | **Papua** | **Betela** | **Nikao** |
| *Phalacroma rapa* | 24 | 21 | 95 | 127 | 30 | 27 |
| *Gambierdiscus australes* | 1580 | 4960 | 1651 | 15362 | 9331 | 17334 |
| *Gambierdiscus honu* | 1409 | 288 | 271 | 3629 | 487 | 1714 |
| *Gambierdiscus caribaeus* | 11 | 16 | 0 | 0 | 1530 | 0 |
| *Gambierdiscus carpenteri* | 52645 | 6278 | 4764 | 8791 | 0 | 2599 |
| *Gambierdiscus pacificus* | 1519 | 958 | 733 | 2072 | 1912 | 2486 |
| *Gambierdiscus polynesiensis* | 4 | 908 | 55 | 306 | 8 | 43 |
| *Gambierdiscus toxicus* | 1 | 0 | 0 | 35 | 0 | 20 |
| *Fukuyoa yasumotoi* | 3 | 0 | 0 | 96 | 0 | 130 |
| *Gambierdiscus cheloniae* | 1725 | 242 | 218 | 981 | 161 | 218 |
| *Alexandrium andersonii* | 63 | 308 | 1127 | 9 | 115 | 0 |
| *Alexandrium insuetum* | 0 | 42 | 20 | 9 | 0 | 51 |
| *Gonyaulax* sp. | 16 | 38 | 18 | 0 | 0 | 0 |
| Gonyaulacaceae unclassified | 1 | 21 | 34 | 0 | 53 | 106 |
| Gonyaulacales unclassified | 126 | 46 | 17 | 107 | 0 | 0 |
| *Amphidinium carterae* | 165 | 421 | 281 | 361 | 98 | 700 |
| *Amphidinium gibbosum* | 0 | 188 | 0 | 126 | 0 | 0 |
| *Amphidinium massartii* | 1216 | 1507 | 1523 | 1368 | 333 | 2256 |
| *Amphidinium* sp. | 0 | 21 | 50 | 0 | 0 | 18 |
| *Amphidinium steinii* | 0 | 47 | 39 | 24 | 19 | 89 |
| *Amphidinium thermaeum* | 478 | 1534 | 1007 | 2573 | 125 | 2320 |
| *Amphidinium trulla* | 15 | 0 | 26 | 0 | 14 | 48 |
| *Cochlodinium polykrikoides* | 56 | 139 | 301 | 10 | 38 | 0 |
| *Gymnodinium dorsalisulcum* | 1734 | 365 | 717 | 355 | 649 | 1237 |
| *Gyrodinium dominans* | 0 | 0 | 46 | 127 | 83 | 16 |
| *Lepidodinium chlorophorum* | 0 | 0 | 0 | 0 | 0 | 12 |
| Gymnodiniaceae unclassified | 1011 | 1219 | 1334 | 0 | 323 | 3453 |
| Gymnodiniales unclassified | 37 | 0 | 22 | 1491 | 0 | 0 |
| *Noctiluca scintillans* | 260 | 110 | 303 | 147 | 122 | 251 |
| *Heterocapsa triquetra* | 0 | 80 | 341 | 0 | 0 | 0 |
| Heterocapsaceae unclassified | 0 | 0 | 12 | 0 | 0 | 0 |
| *Coolia canariensis* | 10 | 22 | 9 | 38 | 14 | 20 |
| *Ostreopsis lenticularis* | 5080 | 3681 | 5292 | 3892 | 2795 | 2163 |
| Ostreopsidaceae unclassified | 2617 | 10967 | 20183 | 1586 | 3535 | 2162 |
| *Durinskia baltica* | 177 | 340 | 544 | 47 | 196 | 1123 |
| Peridiniaceae unclassified | 0 | 0 | 0 | 0 | 0 | 11 |
| Peridiniales unclassified | 24 | 31 | 97 | 22 | 24 | 39 |
| *Prorocentrum emarginatum* | 75 | 214 | 72 | 476 | 52 | 292 |
| *Prorocentrum lima* | 37 | 0 | 32 | 22 | 0 | 50 |
| *Prorocentrum micans* | 111 | 684 | 2001 | 707 | 1602 | 473 |
| *Prorocentrum sculptile* | 12 | 19 | 18 | 125 | 73 | 378 |
| *Prorocentrum* sp. | 28 | 29 | 86 | 25 | 62 | 56 |
| Prorocentrales unclassified | 0 | 721 | 0 | 815 | 1399 | 0 |
| Prorocentraceae unclassified | 498 | 0 | 1318 | 0 | 0 | 869 |
| *Biecheleria cincta* | 0 | 0 | 12 | 0 | 0 | 0 |
| *Polarella* sp. | 51 | 398 | 373 | 0 | 32 | 74 |
| *Symbiodinium* sp. | 13 | 18 | 34 | 0 | 0 | 0 |
| *Symbiodinium* sp. type A | 0 | 45 | 74 | 11 | 13 | 76 |
| Symbiodiniaceae unclassified | 122 | 458 | 606 | 258 | 218 | 723 |
| Suessiales unclassified | 95 | 238 | 144 | 17 | 23 | 69 |
