# Supplementary material for: Risk of ciguatoxins is shaped by Gambierdiscus community structure
Source: PLoS One. 2026 Jan 29;21(1):e0341899. doi: 10.1371/journal.pone.0341899 (PMC12854468; doi:10.1371/journal.pone.0341899)
Supplement: S4 Table — NT: not tested for quantification; ND: not detected. (DOCX) [file pone.0341899.s004.docx]

**Supplementary Table 4.** Ciguatoxin (CTX) results for viscera and flesh samples from *Ctenochaetus striatus* collected from each of the sites around Rarotonga, Cook Islands for both liquid chromatography with tandem mass spectrometry (LC-MS/MS; CTX3B and CTX3C data shown) and the neuroblastoma cell-based assay (CBA-N2a) with screening results of CTX-like activity ranged in three categories (negative, suspect, positive) and quantitative results in ng CTX3C equivalents/g of tissue. NT: not tested for quantification; ND: not detected.

|  |  |  |  | **CBA-N2a** | | **LC-MS** | |
| --- | --- | --- | --- | --- | --- | --- | --- |
| **Cook Islands/Common name** | **Scientific name** | **Matrix** | **Replicate** | **CTX-like activity** | **CTX content  (ng CTX3C eq./g)** | **CTX3B (ng/g)** | **CTX3C (ng/g)** |
| Maito/Striated surgeonfish | *Ctenochaetus striatus* | Flesh | Site Muri - 1 | positive | 0.58 ± 0.04 | Detected | ND |
|  |  |  | Site Muri - 2 | positive | NT | Detected | ND |
|  |  |  | Site Muri - 3 | positive | NT | 1.92 | ND |
|  |  |  | Site Muri - 4 | suspect | NT | <1 | ND |
|  |  |  | Site Muri - 5 | positive | NT | Detected | ND |
|  |  |  | Site Titioki – 1 | positive | 0.21 ± 0.01 | Detected | ND |
|  |  |  | Site Titioki – 2 | positive | NT | Detected | ND |
|  |  |  | Site Titioki – 3 | positive | NT | 1.12 | ND |
|  |  |  | Site Titioki – 4 | positive | NT | Detected | ND |
|  |  |  | Site Titioki – 5 | positive | NT | Detected | ND |
|  |  |  | Site Titikaveka – 1 | suspect | NT | Detected | ND |
|  |  |  | Site Titikaveka – 2 | positive | 0.19 ± 0.01 | 0.953333333 | ND |
|  |  |  | Site Titikaveka – 3 | positive | NT | 9.8 | 1.5 |
|  |  |  | Site Titikaveka – 4 | positive | NT | 2.146666667 | ND |
|  |  |  | Site Titikaveka – 5 | positive | NT | 1.32 | ND |
|  |  |  | Site Papua - 1 | positive | 0.14± 0.01 | ND | ND |
|  |  |  | Site Papua - 2 | suspect | NT | Detected | ND |
|  |  |  | Site Papua - 3 | suspect | NT | Detected | ND |
|  |  |  | Site Papua - 4 | suspect | NT | Detected | ND |
|  |  |  | Site Papua - 5 | positive | NT | ND | ND |
|  |  |  | Site Nikao - 1 | positive | 0.08 ± 0.01 | ND | ND |
|  |  | Viscera | Site Muri composite | positive | 1.97 ± 0.28 | 6.3 | Detected |
|  |  |  | Site Titioki composite | positive | 4.55 ± 0.16 | 11.43333333 | Detected |
|  |  |  | Site Titikaveka composite | positive | 3.65 ± 0.30 | 9.156666667 | Detected |
|  |  |  | Site Papua composite | positive | 4.19 ± 0.28 | 2.7 | 2.7 |
