# Supplementary material for: Risk of ciguatoxins is shaped by Gambierdiscus community structure
Source: PLoS One. 2026 Jan 29;21(1):e0341899. doi: 10.1371/journal.pone.0341899 (PMC12854468; doi:10.1371/journal.pone.0341899)
Supplement: S5 Table — ND = not detected; Pacific ciguatoxins = CTX1B, CTX3C; CTX3B. ND = not detected, NT = not tested for quantification. (DOCX) [file pone.0341899.s005.docx]

**Supplementary Table 5.** Ciguatoxin (CTX) results for flesh and liver samples from fish species collected from each of the sites around Rarotonga, Cook Islands for both liquid chromatography with tandem mass spectrometry (LC-MS/MS; CTX3B and CTX3C data shown) and the neuroblastoma cell-based assay (CBA-N2a; CTX3C equivalents). ND = not detected; Pacific ciguatoxins = CTX1B, CTX3C; CTX3B. ND = not detected, NT = not tested for quantification.

|  |  |  | **CBA-N2a** | | **LC-MS/MS** | |
| --- | --- | --- | --- | --- | --- | --- |
| **Cook Islands/Common name** | **Scientific name** | **Matrix** | **CTX-like activity** | **CTX content (ng CTX1B eq./g)** | **CTX3B** | **CTX3C** |
| Pātuki roi/Peacock grouper | *Cephalopholis argus* | Flesh | positive | NT | ND | ND |
|  |  | Liver | positive | NT | ND | ND |
| U'u'/Parrotfish | *Chlororus frontalis* | Flesh | positive | NT | Detected | ND |
|  |  | Liver | suspect | NT | ND | ND |
| Pātuki paru/Hexagon Grouper | *Epinephelus hexagonatus* | Flesh | positive | 0.04 ± 0.00 | Detected | ND |
|  |  | Viscera | positive | 1.29 ± 0.16 | 1.4 | ND |
| Kanae/Mullet | *Crenimugil crenilabis* | Flesh | positive | NT | 1.52 | ND |
|  |  | Liver | positive | NT | ND | ND |
| Ka‘uru/Manybar goatfish | *Parupeneus multifasciatus* | Flesh | suspect | NT | ND | ND |
|  |  | Liver | positive | NT | 5.45 | 2.2 |
| Ka‘uru/Doublebar goatfish | *Parupeneus insularis* | Flesh | suspect | NT | ND | ND |
|  |  | Liver | positive | NT | 11.88 | 4.3 |
| Ka‘uru/Dash-and-dot goatfish | *Parupeneus barberinus* | Flesh | positive | NT | ND | ND |
|  |  | Liver | positive | NT | Detected | ND |
| Vete/Yellowstripe goatfish | *Mulloidichthys flavolineatus* | Flesh | positive | NT | ND | ND |
|  |  | Liver | positive | NT | ND | ND |
| Katoti/Dusky damsel | *Stegastes nigricans* | Flesh | positive | NT | Detected | Detected |
|  |  | Viscera | positive | NT | Detected | Detected |
