# Supplementary material for: Risk of ciguatoxins is shaped by Gambierdiscus community structure
Source: PLoS One. 2026 Jan 29;21(1):e0341899. doi: 10.1371/journal.pone.0341899 (PMC12854468; doi:10.1371/journal.pone.0341899)
Supplement: S6 Table — Fish samples were homogenized whole and five fish were pooled from each location. ND = not detected. (DOCX) [file pone.0341899.s006.docx]

**Supplementary Table 6.** Ciguatoxin (CTX) results for *Chrysiptera glauca* collected from each of the sites around Rarotonga, Cook Islands for both liquid chromatography with tandem mass spectrometry (LC-MS/MS; CTX3B and CTX3C data shown) and using the neuroblastoma cell-based assay (CBA-N2a) with screening results of CTX-like activity ranged in three categories (negative, suspect, positive). Fish samples were homogenized whole and five fish were pooled from each location. ND = not detected.

|  |  |  |  | **CBA-N2a** | **LC-MS/MS** | |
| --- | --- | --- | --- | --- | --- | --- |
| **Cook Islands/Common name** | **Scientific name** | **Matrix** | **Replicate** | **CTX-like activity** | **CTX3B (ng/g)** | **CTX3C (ng/g)** |
| Katoti/Grey damsel | *Chrysiptera glauca* | whole | Site Muri | Negative | ND | ND |
|  |  |  | Site Tikioki | Suspect | ND | ND |
|  |  |  | Site Titikaveka | Negative | ND | ND |
|  |  |  | Site Papua | Negative | ND | ND |
|  |  |  | Betela | Negative | ND | ND |
|  |  |  | Nikao | Negative | ND | ND |
