# Supplementary material for: Risk of ciguatoxins is shaped by Gambierdiscus community structure
Source: PLoS One. 2026 Jan 29;21(1):e0341899. doi: 10.1371/journal.pone.0341899 (PMC12854468; doi:10.1371/journal.pone.0341899)
Supplement: S1 Fig — Sequences in bold represent the consensus sequence from all reads of each taxonomic assignment. Values at nodes represent Bayesian posterior probability support. Scale bar is substitutions per site. (DOCX) [file pone.0341899.s007.docx]

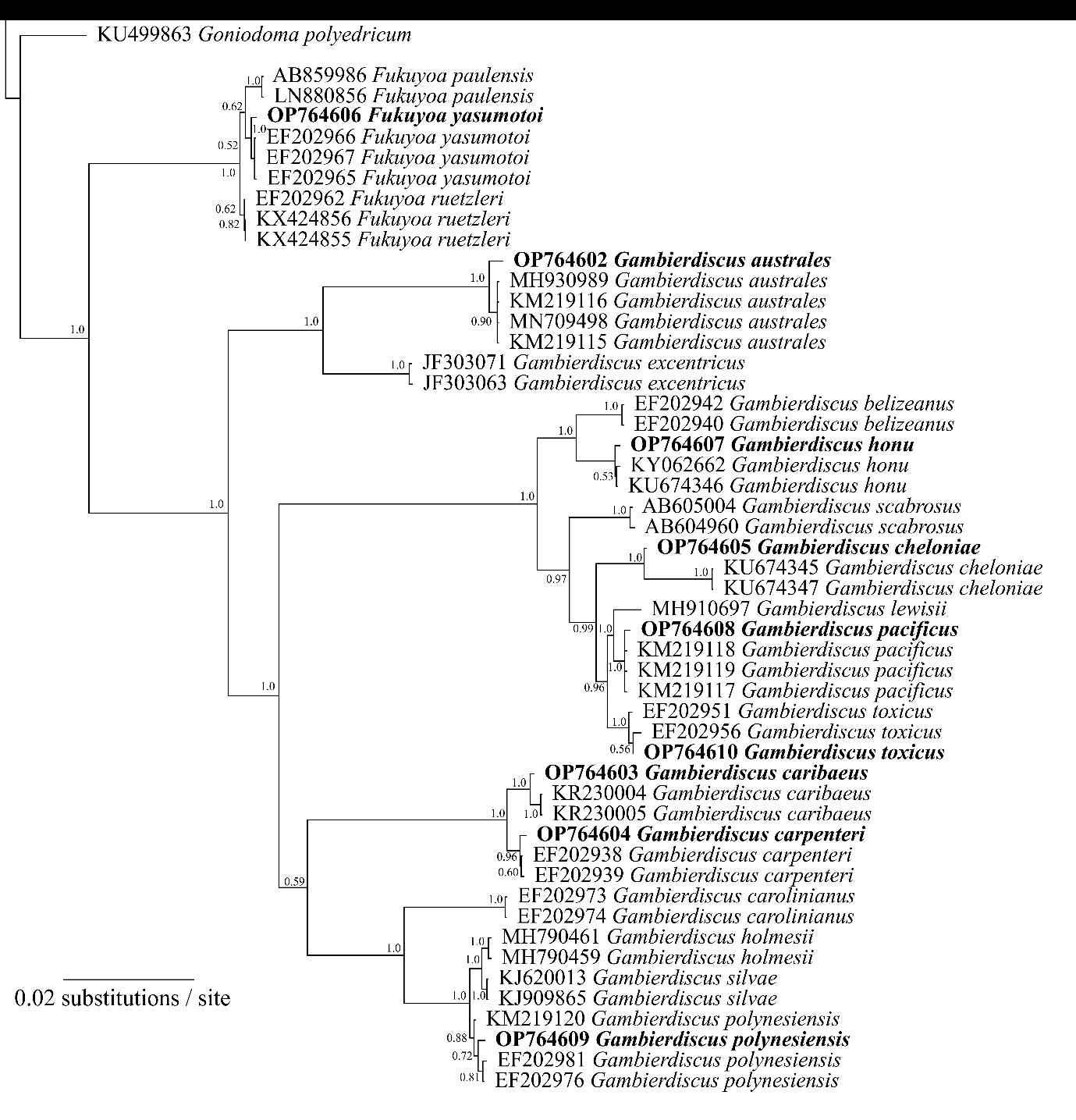


**Supplementary Figure 1.** Phylogenetic analysis of large subunit ribosomal RNA (LSU) sequences obtained from the high-throughput sequencing (HTS) metabarcoding from sampling sites around Rarotonga (Cook Islands). Sequences in bold represent the consensus sequence from all reads of each taxonomic assignment. Values at nodes represent Bayesian posterior probability support. Scale bar is substitutions per site.
