# Supplementary material for: Risk of ciguatoxins is shaped by Gambierdiscus community structure
Source: PLoS One. 2026 Jan 29;21(1):e0341899. doi: 10.1371/journal.pone.0341899 (PMC12854468; doi:10.1371/journal.pone.0341899)
Supplement: S2 Fig — A. Linear regression analysis showing the relationship between the length of Ctenochaetus striatus and the weight of its otolith. B. Linear regression analysis showing the relationship between the length of C. striatus and the age estimate of its otolith. (DOCX) [file pone.0341899.s008.docx]

A

B

**Supplementary Figure 2.** A. Linear regression analysis showing the relationship between the length of *Ctenochaetus striatus* and the weight of its otolith. B. Linear regression analysis showing the relationship between the length of *C. striatus* and the age estimate of its otolith.
