# Supplementary material for: Risk of ciguatoxins is shaped by Gambierdiscus community structure
Source: PLoS One. 2026 Jan 29;21(1):e0341899. doi: 10.1371/journal.pone.0341899 (PMC12854468; doi:10.1371/journal.pone.0341899)
Supplement: S3 Fig — Values at nodes represent Bayesian posterior probability support. Scale bar is substitutions per site. (DOCX) [file pone.0341899.s009.docx]

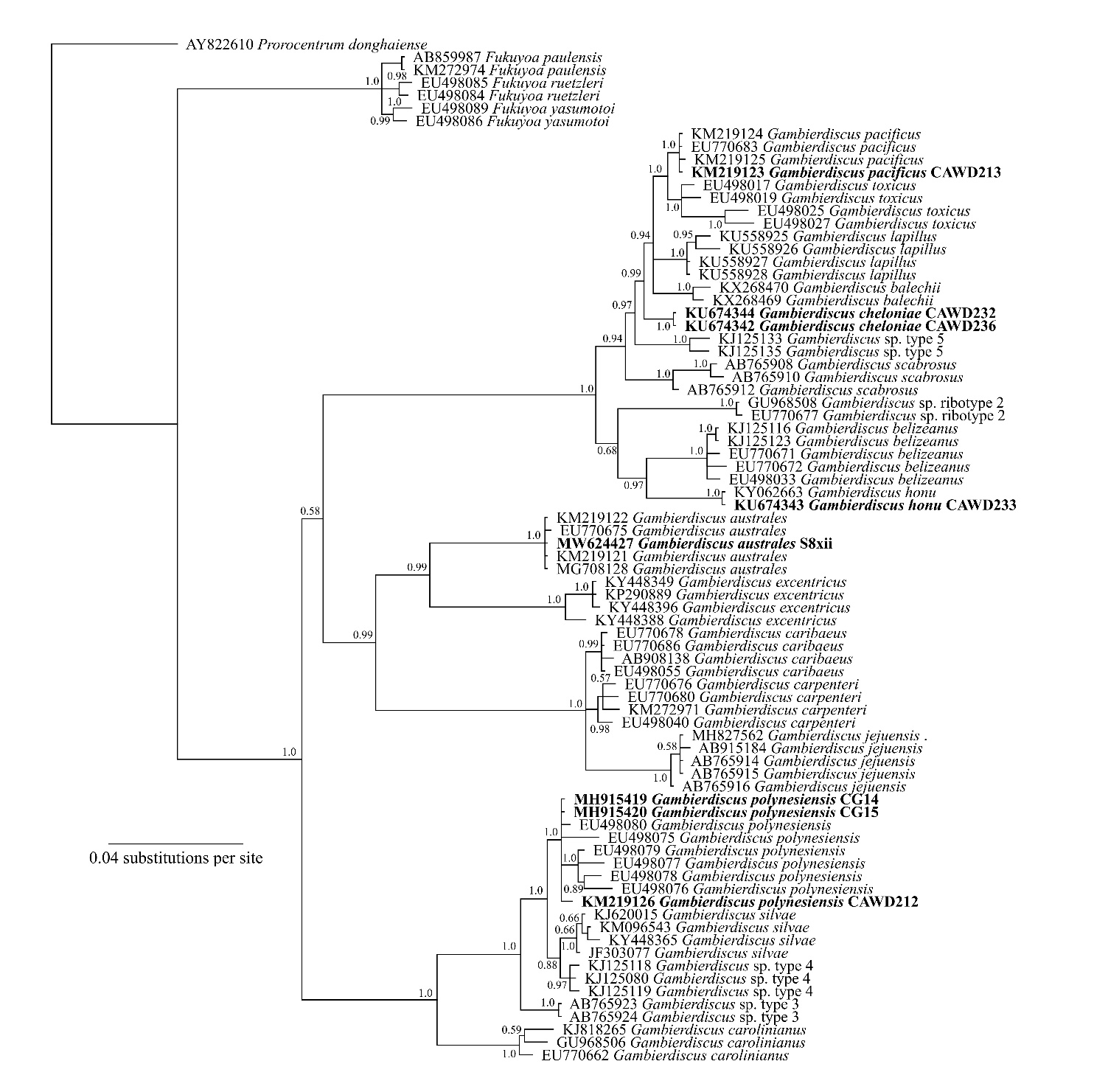


**Supplementary Figure 3.** Phylogenetic analyses using the large subunit ribosomal RNA (LSU) (D1-D3 region) sequences from *Gambierdiscus* strains isolated during this study from sampling sites around Rarotonga (Cook Islands) using Bayesian analyses. Values at nodes represent Bayesian posterior probability support. Scale bar is substitutions per site.
