# Supplementary material for: Risk of ciguatoxins is shaped by Gambierdiscus community structure
Source: PLoS One. 2026 Jan 29;21(1):e0341899. doi: 10.1371/journal.pone.0341899 (PMC12854468; doi:10.1371/journal.pone.0341899)
Supplement: S4 Fig — (DOCX) [file pone.0341899.s010.docx]

**
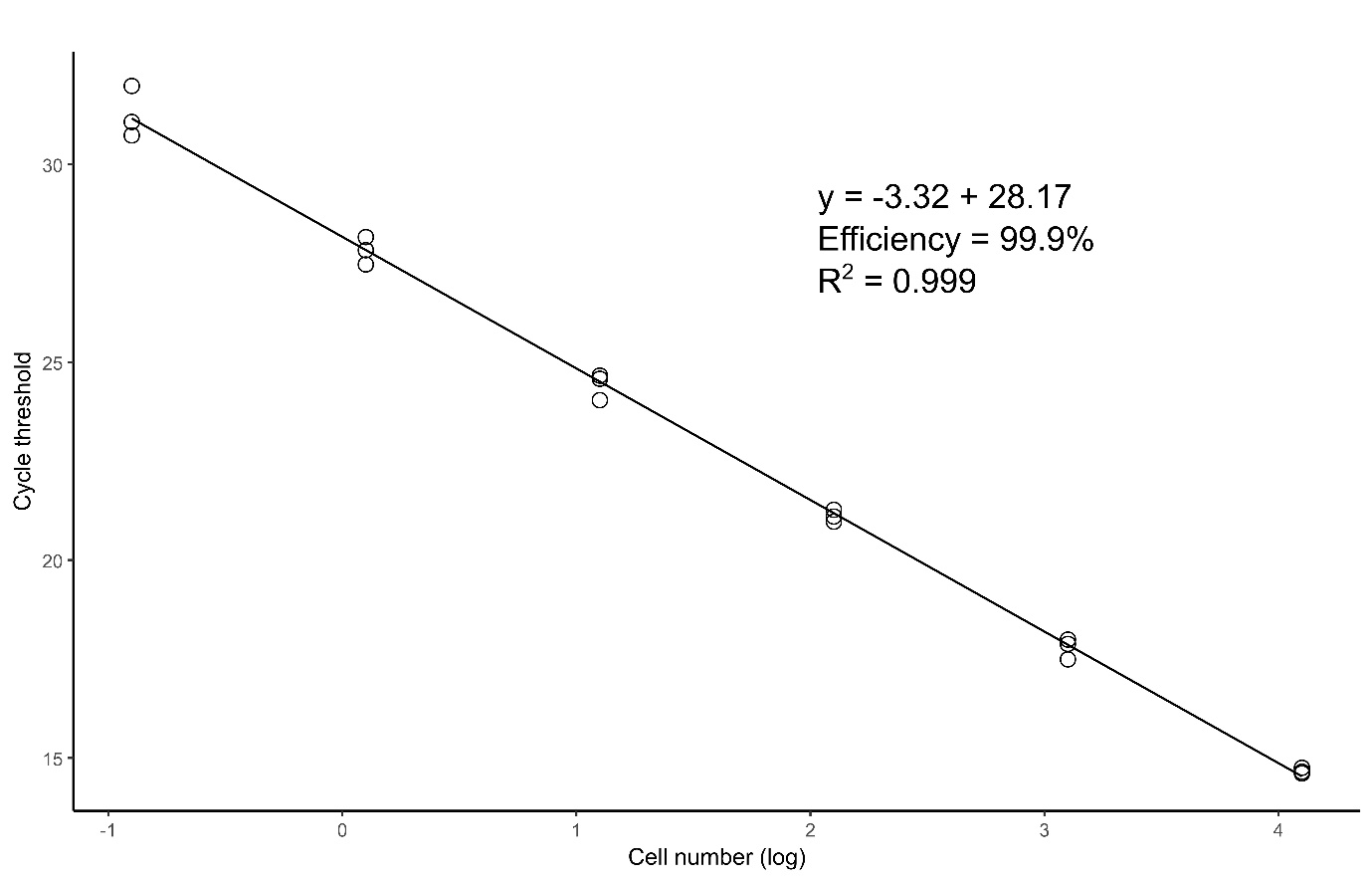
**

**Supplementary Figure 4.** Standard curve for the *Gambierdiscus polynesiensis* real-time PCR assay constructed with 10-fold serial dilutions of genomic DNA extracts from available *G. polynesiensis* culture (CG14).
